# Quantitative analysis of genomic element interactions by molecular colony technique

**DOI:** 10.1093/nar/gkt1322

**Published:** 2013-12-24

**Authors:** Alexey A. Gavrilov, Helena V. Chetverina, Elina S. Chermnykh, Sergey V. Razin, Alexander B. Chetverin

**Affiliations:** ^1^Group of Genome Spatial Organization, Institute of Gene Biology of the Russian Academy of Sciences, Moscow 119334, Russia, ^2^Laboratory of Viral RNA Biochemistry, Institute of Protein Research of the Russian Academy of Sciences, Pushchino, Moscow Region 142290, Russia, ^3^Laboratory of Cell Proliferation Problems, Koltzov Institute of Developmental Biology of the Russian Academy of Sciences, Moscow 119334, Russia, ^4^Laboratory of Structural and Functional Organization of Chromosomes, Institute of Gene Biology of the Russian Academy of Sciences, Moscow 119334, Russia and ^5^Faculty of Biology, M.V. Lomonosov Moscow State University, Moscow 119992, Russia

## Abstract

Distant genomic elements were found to interact within the folded eukaryotic genome. However, the used experimental approach (chromosome conformation capture, 3C) enables neither determination of the percentage of cells in which the interactions occur nor demonstration of simultaneous interaction of >2 genomic elements. Each of the above can be done using in-gel replication of interacting DNA segments, the technique reported here. Chromatin fragments released from formaldehyde–cross-linked cells by sodium dodecyl sulfate extraction and sonication are distributed in a polyacrylamide gel layer followed by amplification of selected test regions directly in the gel by multiplex polymerase chain reaction. The fragments that have been cross-linked and separate fragments give rise to multi- and monocomponent molecular colonies, respectively, which can be distinguished and counted. Using in-gel replication of interacting DNA segments, we demonstrate that in the material from mouse erythroid cells, the majority of fragments containing the promoters of active β-globin genes and their remote enhancers do not form complexes stable enough to survive sodium dodecyl sulfate extraction and sonication. This indicates that either these elements do not interact directly in the majority of cells at a given time moment, or the formed DNA–protein complex cannot be stabilized by formaldehyde cross-linking.

## INTRODUCTION

The 3D structure of the eukaryotic genome and its role in the regulation of gene expression has recently gained much attention (1–12). Although the mechanism of the activity of transcription enhancers is far from being clear, most of the current models postulate that an enhancer directly interacts with the target promoter, whereas the segment of the chromatin fiber that separates the promoter and the enhancer is looped out (13–15).

A single enhancer may activate several promoters. For example, in erythroid cells of the adult lineage, the mouse β-globin locus control region (LCR) stimulates the expression of both the β-major and β-minor globin genes. The promoters of these genes are located at a distance of 14 kb from each other and could not simultaneously interact with the same LCR if only a single loop was formed. Therefore, it was proposed that LCR interacts simultaneously with several target promoters in an active chromatin hub (ACH), a multicomponent complex from which several chromatin segments are looped out. Although the ACH model (16,17) is widely accepted by scientific community (6,18–20), it remains a hypothesis because the 3C analysis only allows relative frequencies of pairwise interactions between distant chromosome elements to be determined (21) and cannot ascertain whether two or more pairwise interactions occur simultaneously at a single location in the same cell. The same results could also be explained by alternate associations of an enhancer with each of the activated promoters (22,23). Accordingly, in each single moment different pairwise enhancer–promoter interactions might occur in different subpopulation of the cells. Furthermore, either 3C or other methods based on the proximity ligation (24–27) cannot provide for estimating the proportion of cells in which two particular DNA sequences interact (28,29). Therefore, even the most typical spatial configuration of a genomic locus remains unknown. Quantitative analysis of the 3C data is complicated by the fact that each end of a restriction fragment may be ligated to any of the ends of an unknown number of other fragments present in the same chromatin complex or located in proximity. Among the current methods, only fluorescence *in situ* hybridization (FISH) can detect multicomponent complexes of remote chromosomal fragments within the cell nucleus. However, the resolution of this approach is limited. Although recently, FISH was reported to be able of resolving chromosomal elements spaced by 50 kb (30) or even less (31), FISH is rarely used to analyze the configuration of genomic loci <150 kb (32).

Here, we report protocol INGRID (IN-Gel Replication of Interacting DNA segments), which enables a direct identification of multicomponent DNA complexes and determination of the percentage of complexes of a given type. The key feature of the INGRID protocol is spreading cross-linked chromatin fragments over large area of a polyacrylamide gel layer followed by visualization of separate and associated elements in the form of, respectively, mono- and multicomponent molecular colonies generated during in-gel amplification of selected DNA fragments (33). Using this approach, we show that in the mouse erythroid cells, cross-linked complexes containing promoters of the two active β-globin genes (Pβ^maj^ and Pβ^min^) and the LCR, as well as the respective pairwise combinations, account for <3% of each of the constituent DNA fragments. These results suggest that the ACH model needs to be refined.

## MATERIALS AND METHODS

### INGRID assay

Fetal livers were isolated from a day 14.5 mouse embryo, disrupted by pipetting in Dulbecco’s modified Eagle’s medium supplemented with 10% FBS (fetal bovine serum) and passed through a 30-µm cell strainer to produce a single-cell suspension.

To prepare formaldehyde–cross-linked chromatin fragments, 5 × 10^7^ cells were incubated in PBS (phosphate-buffered saline) supplied with 10% FBS and 1% formaldehyde for 10 min at room temperature, and then the reaction was stopped by the addition of glycine to 0.125 M. The fixed cells were washed with ice-cold PBS supplied with 10% FBS and, when desired, sorted on anti-Ter119 microbeads using the magnetic-activated cell sorting (MACS) separation system (Miltenyi Biotec) according to the manufacturer’s protocol. To release cross-linked chromatin fragments, 3 × 10^6^ cells were sonicated in 0.6 ml of an ice-cold sonication buffer [50 mM Tris pH 8.0, 50 mM NaCl, 5 mM EDTA, 1% SDS (sodium dodecyl sulfate) and a protease inhibitors cocktail (Complete Mini, Roche)] in 1 pulse of 7 or 15 s using VirTis VirSonic 100 sonicator at medium power (setting 7). The non-solubilized material was removed by centrifugation at 16 000*g* for 20 min at room temperature, and the supernatant was subjected to in-gel polymerase chain reaction (PCR) after first 150-fold dilution with a PCR buffer devoid of KCl (to prevent SDS precipitation) and then further 100- to 200-fold dilution with a PCR cocktail containing all reaction components except the DNA template (see later in the text).

For HindIII digestion of solubilized chromatin fragments, 100 µl of the supernatant was diluted 6-fold with 1.2× restriction buffer 2 (New England Biolabs) supplemented with 2% Triton X-100, and the solution was incubated for 1 h at 37°C with shaking to sequester the SDS. The DNA was digested by overnight incubation with 100 U of HindIII (New England Biolabs) at 37°C with shaking.

To prepare doubly cross-linked chromatin fragments, the cells were incubated in PBS supplied with 10% FBS, 10% dimethyl sulfoxide (DMSO) and 1.5 mM [ethylene glycol bis(succinimidylsuccinate) (EGS)] for 30 min at room temperature followed by addition of formaldehyde to 1% and incubation for 10 min at room temperature, after which the cells were treated as described earlier in the text, with the only difference that sonication was performed in 2 pulses of 10 s each at high power (setting 15).

To prepare soluble fraction of the 3C material, the cells were treated according to the 3C protocol (17,29) up to the 1.6% SDS extraction step. Then the insoluble material was removed by centrifugation (16 000*g* for 20 min at room temperature) (34).

The in-gel PCR was carried out as described (35) using 0.4-mm-thick, 14-mm-diameter polyacrylamide gels (7% acrylamide, 0.07% bisacrylamide) that were cast in wells drilled in microscopy slides, washed and dried (36). Before the experiment, each well was filled up, without allowing air bubbles, under a glass coverslip placed on a ring of a mineral oil (2.5 µl) applied around the well, with 60–65 µl of a PCR cocktail containing buffer [50 mM Tris (pH 8.6), 50 mM KCl, 1.5 mM MgCl_2_, 0.1% Tween 20 and 1 mg/ml bovine serum albumin], 0.2 mM each of dNTPs, 0.3 µM each of the primers, 0.04 µM each of the molecular beacon probes, 3 U of Hot Start (antibody blocked) Taq DNA polymerase (SibEnzyme) and the appropriate DNA template. The wells were sealed with a piece of adhesive PCR foil (Eppendorf), in which a round window of a diameter equal to that of the well was cut off beforehand, and incubated at 4°C for 1.5 h, during which time the gel absorbed all of the liquid. Swollen gels were subjected to PCR as follows: initial heating for 3 min at 94°C (which served for the cross-link reversal and polymerase activation) followed by up to 41 PCR cycles (melting for 10 s at 94°C, primer annealing for 30 s at 55°C and template copying for 30 s at 72°C). PCR was carried out in a PCR machine with a flat-bed heating block. The growth of DNA colonies was monitored by taking the slides with amplification gels out of the thermocycler in a middle of the annealing step at a specified cycle number and scanning them three times at a 50-µm resolution with a confocal microarray reader (ScanArray Express, PerkinElmer), to detect the fluorescence of FAM (using a 488-nm blue laser and a 508-nm emission filter), Cyanine 3 (550-nm green laser and 570-nm emission filter) and Cyanine 5 (649-nm red laser 670-nm emission filter), in the indicated order, after which PCR was resumed.

The gel images obtained by detecting the fluorescence of FAM, Cy3 and Cy5 were processed with Adobe Photoshop after having been artificially colored blue, green and red, respectively, using Leica LAS AF Lite software and, where indicated, merged using ImageJ software.

The sequences of primers and molecular beacons are presented in Table 1. Molecular beacon probes were designed using Quikfold application of the DINAMelt web server for nucleic acid melting prediction (http://mfold.rna.albany.edu/?q=DINAMelt/Quickfold).

**Table 1.** Sequences of primers and molecular beacon probes used for the INGRID analysis

Test region	Amplicon size, bp	Primer/molecular beacon set (5′–3′)
HS-62	258	S GGGCTATGCTGCCATGTGAT
		A/S CTTGTGTTAAGTTGGAGTGGGAA
		MB FAM-cccgctACCTGCCATACAACCGAGAACTCCagcggg-BHQ1

−42 region	279	S GAACTCTGCACTCATCACATTGG
		A/S AGTATGTTGGCAATATCTCAAAGGT
		MB Cy3-cgcgctTGTAATACAGACTAATTCTAGGGCagcgcg-BHQ2

Upstream of HS5	260	S ACTTCTCCTGTTGGTGTGGCA
		A/S AGGAAACAAAATCACCTGCACA
		MB Cy3-cgcgcTGTGGTCATGGTCCTGTTAGTCTCAAGgcgcg-BHQ2

HS5	250	S AGTGAAGGATGAGAACTTGAATGC
		A/S CTCTACCATGAAAATGACGCCTA
		MB FAM-ccggctGAGGCGTTTTCACCACTAGAGGGagccgg-BHQ1

Pβ^maj^	293	S TCAGTAGTTGATTGAGCAAATGTGTT
		A/S CTATGTCAGAAGCAAATGTGAGGAG
		MB Cy3-cgcgctAAGCCTGATTCCGTAGAGCCACACCagcgcg-BHQ2

Pβ^min^	227	S CAAGGATAAGAACAGACACTACTCAGA
		A/S GAATCAGAAGCAAACGTAAGAAGC
		MB Cy5-cgccgCACCCTGTGTAGATATGGTTGTCAcggcg-BHQ3

Chr3	313	S AGCAAATGTGTCTCCCAGATGTT
		A/S GCTGATGAGAGTTCACCACTACCA
		MB Cy3-cgcgcTGTCTCTGTTGGAGGGCTCAGGAAGgcgcg-BHQ2

S, sense primers; A/S, antisense primers (in regard to the direction of transcription of the β-globin genes); MB, molecular beacon probes (stem parts are shown in lowercase letters; central parts complementary to target DNA sequences are underlined).

## RESULTS

### Principle of the INGRID protocol

**Figure 1.**
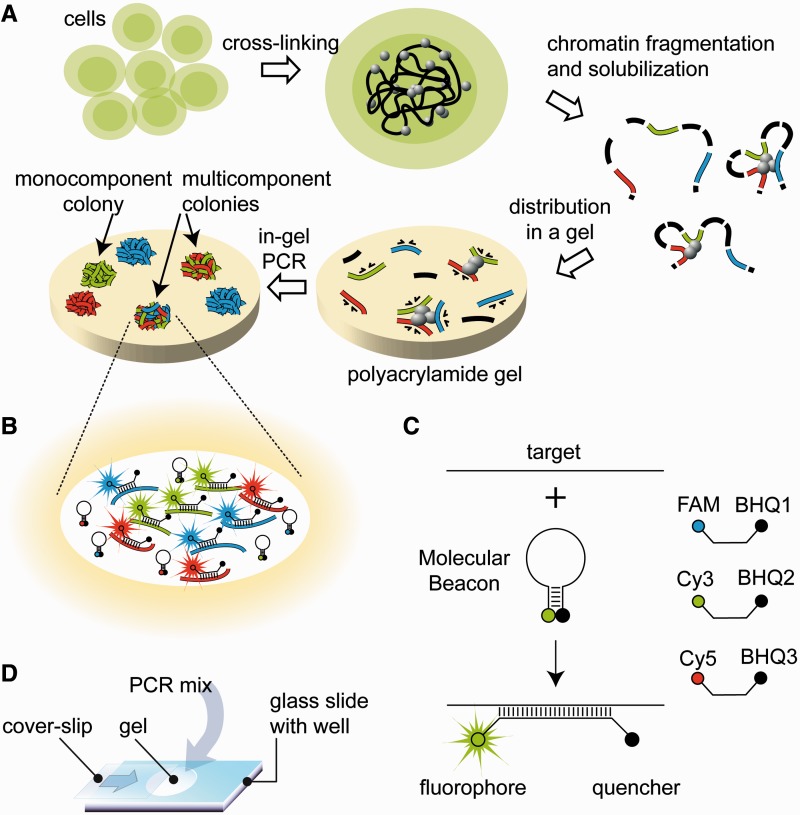
Outline of the INGRID procedure. (**A**) The primary steps of the procedure. After fixation with formaldehyde, cells are lysed and the chromatin is fragmented (by sonication, restriction enzyme digestion, etc.). The released chromatin segments are randomly distributed in a layer of a polyacrylamide gel (PAAG), and the DNA fragments of interest (test amplicons) are amplified in the gel. The PCR products remain close to the original template because of the absence of convection and restricted diffusion in PAAG and form molecular colonies. The formation of a multicomponent colony comprising several chromosome elements indicates that they are juxtaposed in the cell. (**B**) The enlarged scheme of a multicomponent (ternary) molecular colony illustrating the principle of the sequence-specific visualization of amplicons using the molecular beacon probes (38). (**C**) Principle of the molecular beacon action. These are oligonucleotides labeled at opposite ends with a fluorophore and its quencher and capable of folding into a hairpin due to the presence of complementary terminal sequences. The looped central region of the oligonucleotide is complementary to a target sequence. Hybridization of the probe to its target displaces the quencher from the fluorophore, resulting in increased fluorescence. The fluorophore/quencher combinations used in this study are shown on the right. (**D**) The setup of in-gel RCR.

The concept behind INGRID is shown in Figure 1. The principal difference of INGRID from 3C and derivative methods (24–27,37) is that cross-linked chromatin fragments released from nuclei are not ligated. Instead, they are distributed in a layer of a polyacrylamide gel (Figure 1), and the DNA fragments of interest (test-amplicons) are PCR-amplified directly in the gel to produce molecular colonies (33,36). The growth of the colonies is monitored in real time (35) using, e.g. molecular beacon probes (38) (Figure 1). The chromatin segments that have been cross-linked are colocalized in the gel and give rise to multicomponent colonies, whereas non–cross-linked segments are distributed in the gel independently of each other and typically produce monocomponent colonies. Thus, INGRID allows individual complexes of DNA elements to be visualized and counted by converting them to multicomponent DNA colonies.

### Applying INGRID to the murine β-globin gene locus

**Figure 2.**
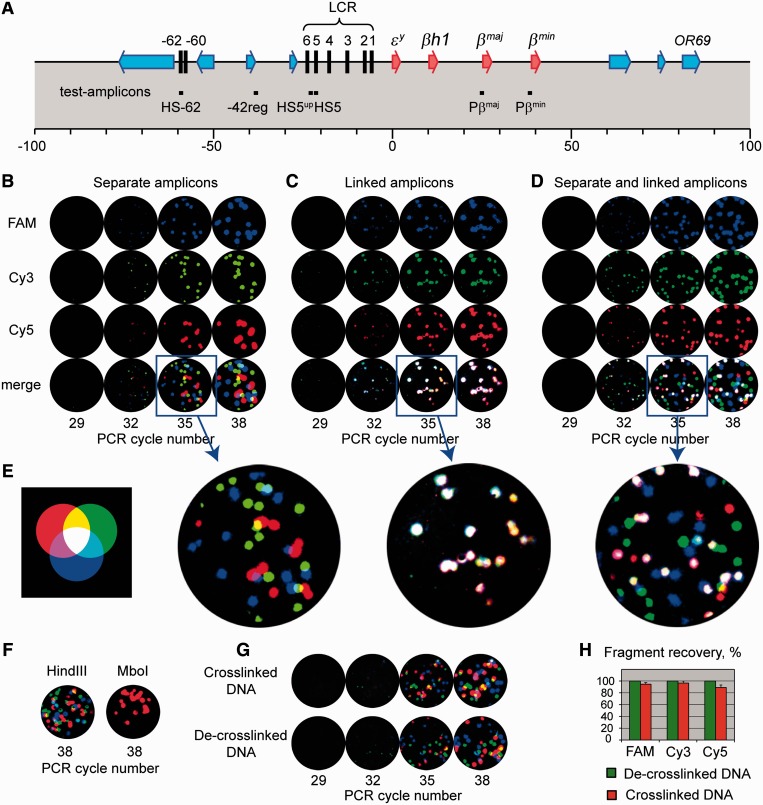
Molecular colonies produced by in-gel PCR amplification of test fragments from the murine β-globin locus. (**A**) Map of the murine β-globin locus showing β-globin genes (red arrows), olfactory receptor genes (OR, blue arrows) and the DNase I hypersensitive sites (HS, black vertical lines). The position of the test amplicons (named as in the text) is shown with black boxes below the map. The scale is in kilobases and according to the mouse genome contig (GenBank entry NT_039433.7). (**B–D**) Detection of DNA colonies using molecular beacons labeled with fluorophores FAM, Cy3 and Cy5, (B) gel containing ∼15 copies each of the sequences separately cloned in plasmids, (C) gel containing ∼15 copies of a plasmid carrying all three of the sequences, (D) gel containing ∼15 copies each of the separately cloned sequences plus ∼15 copies of a plasmid carrying all three of the sequences. (**E**) The color scheme used for the presentation of the gel images. The images obtained on detection of the FAM, Cy3, and Cy5 fluorescence have been artificially colored blue, green and red, respectively; if the signals overlap, the three colors produce a white color, whereas pairwise combinations produce yellow, cyan and magenta. (**F**) Merged images of two gels containing ∼15 copies of a haploid set of the murine genomic DNA digested at the HindIII or MboI sites as indicated. (**G**) Merged images of two gels containing formaldehyde–cross-linked fragments distributed in a gel either directly (cross-linked DNA) or after a standard cross-link reversal and DNA isolation procedure (de–cross-linked DNA). Each gel contained ∼15 copies of the haploid murine genome. (**H**) Histogram showing the results of three independent experiments carried out as in (G) in which six gels with samples of each of the two types were analyzed. The copy number of fragments detected in the experiments with de–cross-linked DNA was taken equal to 100%. Eror bars represent the standard deviation.

To test the INGRID assay, we have studied the spatial organization of the mouse β-globin locus (Figure 2A), which has been extensively characterized with the conventional 3C method (6,16,17,34,39). For the first set of experiments, we selected primers and molecular beacons targeting the DNase I hypersensitive site 5 (HS5) of the LCR and promoters of the major and minor β-globin genes (Pβ^maj^ and Pβ^min^, respectively; see Figure 2A and Table 1), which are thought to be components of an ACH (17). The specificity of amplification was checked in a standard PCR followed by gel electrophoresis of PCR products (Supplementary Figure S1). In preliminary experiments, we checked whether the three selected sequences that are simultaneously amplified in the same gel can individually be monitored using a mixture of molecular beacon probes labeled with fluorophores FAM, Cy3 and Cy5. The three sequences were separately cloned in plasmids, and, on average, 15 copies of each construct were distributed in the gel. Figure 2B demonstrates that the individual colonies of the three types can easily be distinguished. The images obtained on detection of the FAM, Cy3 and Cy5 fluorescence have been artificially colored blue, green and red, respectively. If the signals overlapped, the three colors produced a white color, whereas pairwise combinations produced yellow, cyan and magenta colors (see the color scheme in Figure 2E). The colonies appeared at the 32nd cycle of the in-gel PCR and became perfectly visible after the 35th cycle. Although sometimes colonies of two different colors did partially overlap (see the enlarged image in Figure 2B), we did not observe the overlap of all three colors. In contrast, if a gel was loaded with a plasmid carrying the three target sequences as a triple insertion, each colony hybridized with all three probes and appeared white in the merged image, which is indicative of a multicomponent colony (Figure 2C). Finally, when the three plasmids carrying individual amplicons and the plasmid in which the three amplicons were linked were distributed in the same gel, the mono- and multicomponent colonies were clearly distinguished (Figure 2D). The signal from the individual probes in both mono- and multicomponent colonies became detectable at the same (32nd) PCR cycle, indicating that the amplification of one of the colocalized amplicons did not appreciably affect the amplification of the others. We also examined whether the chosen primers and molecular beacon probes were suitable for the detection of the three segments from the entire mouse genome digested with the restriction endonuclease HindIII. Despite the large excess of irrelevant DNA, each of the three segments replicated well, resulting in approximately the same number of colonies (Figure 2F, left image). Additionally, the absence of false positives was verified by selectively digesting the amplicons that produced the blue and green colonies by taking advantage of the presence of MboI restriction sites in the corresponding segments. The image on the right in Figure 2F shows that only red colonies were formed in the gel in which the MboI-digested fragments of genomic DNA were distributed.

A standard protocol for the reversal of formaldehyde cross-links includes overnight incubation of the sample at 65°C in the presence of proteinase K followed by DNA purification by means of phenol/chloroform extraction and ethanol precipitation (16,21). Such a procedure would be cumbersome with gel-immobilized DNA–protein complexes. To simplify the INGRID protocol, we omitted the cross-link reversal and protein digestion steps of the standard 3C procedure. We reasoned that the initial incubation at 94°C might itself reverse the cross-links, making DNA accessible for subsequent amplification, whereas a substantial dilution of the material applied to a gel would make the DNA isolation unnecessary. To test whether the above modifications would affect the performance of the assay, we compared the yield of molecular colonies in experiments when the cross-linked chromatin fragments were distributed in a gel directly or after having been subjected to the standard cross-link reversal and deproteinization procedures. In each case, the copy numbers of the amplifiable DNA templates were nearly the same (Figure 2G and H).

### Analysis of interaction frequencies of the β-globin gene domain fragments solubilized from cross-linked erythroid cells

The liver of the day 14.5 mouse embryo was used as a source of erythroid cells. In the first set of experiments, the cells were fixed with formaldehyde and further purified by sorting on magnetic beads with tethered antibodies to Ter119, a specific receptor on erythroid cells (28). This allowed us to separate the erythroid Ter119^+^ cells from the non-erythroid (e.g. parenchyma) cells present in the embryonic liver (40). Homogeneity of the cell populations is especially important for the INGRID assay, which allows chromatin interactions to be studied at the level of a single chromosomal locus.

**Figure 3.**
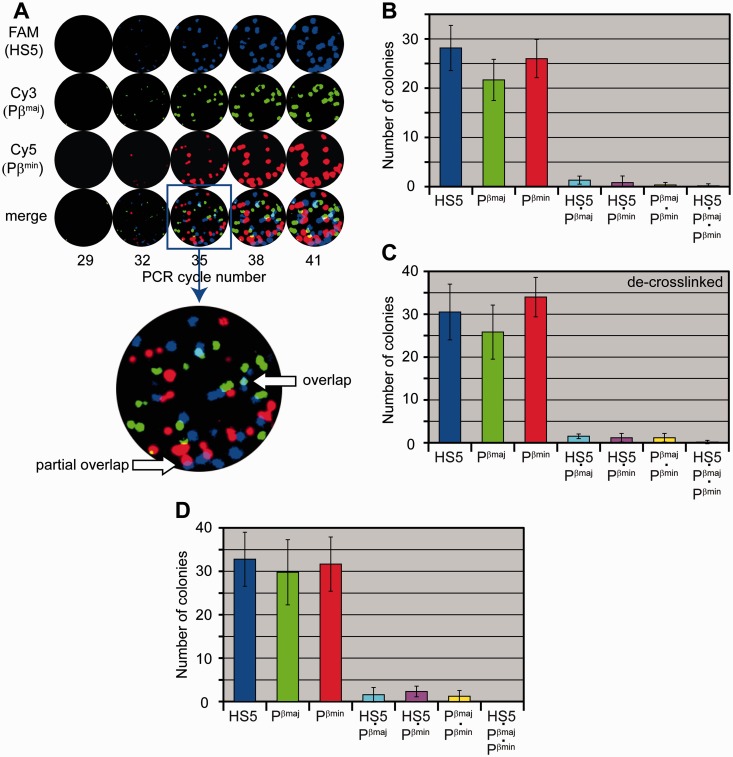
INGRID analysis of the chromatin fragments released from formaldehyde-fixed mouse erythroid (Ter119^+^) cells on (**A–C**) 7-s pulse of sonication or (**D**) 15-s pulse of sonication. (A) Gel containing cross-linked fragments equivalent to ∼30 copies of the haploid murine genome. The arrows on the enlarged gel image show examples of overlapping and partially overlapping colonies. (B–D) Diagrams showing a mean number of colonies per gel calculated on inspection of six gels (B, C) or nine gels (D) containing the material from cross-linked cells (B, D), and the same material after the standard cross-link reversal and DNA isolation procedure (C). In each diagram, from left to right, the first three columns represent the total number of colonies hybridizing with an indicated probe (including monocomponent, binary and ternary colonies), the second three columns represent the number of colonies hybridizing with an indicated pair of probes (binary colonies) and the last column represents the number of colonies hybridizing with all of the three probes used (ternary colonies). Error bars represent the standard deviation. The raw data are presented in Supplementary Table S2.

To release chromatin fragments, Ter119^+^ cells were sonicated in SDS-containing buffer followed by removal of the remaining insoluble material by centrifugation. Although sonication ensures effective solubilization of cross-linked material, it also causes occasional breakage of test amplicons. In a preliminary experiment (Supplementary Figure S2), we have selected conditions of sonication (7-s pulse), which ensured the minimal breakage of test amplicons (<10%, Supplementary Figure S2F) and yet provided for the solubilization of ∼90% of DNA fragments (Supplementary Figure S2A, B and D). After an appropriate dilution (we used the material containing ∼30 copies of a haploid murine genome per gel), the supernatant was subjected to in-gel amplification using primers and molecular beacons targeting LCR HS5, Pβ^maj^ and Pβ^min^. The number of colonies produced (Figure 3A and B) was 10–20% less than could be expected based on the amount of DNA applied to a gel, which might be due to the breakage of amplicons after sonication (Supplementary Figure S2F), incomplete recovery of amplifiable DNA fragments due to omitting the cross-link reversal and protein digestion steps (Figure 2H) or loss of material during manipulations. Still, the portion of amplifiable templates was rather high, which was important for the analysis of colocalization frequencies.

Analysis of the material from Ter119^+^ cells did not reveal overlapping colonies of white color, indicating that all three amplicons are rarely (and perhaps, never) present in the same complex of cross-linked chromatin fragments (Figure 3A). Colonies containing two of the three amplicons in different combinations (binary colonies) were observed, but the percentage of such colonies was relatively low. To account for the rate of accidental overlapping between two or more molecular colonies, a portion of cross-linked chromatin fragments was subjected to the standard cross-link reversal and DNA isolation procedures before the molecular colony analysis so that any ties between DNA fragments would have been eliminated before they were distributed in the gel. Then, taking into consideration only perfectly matching colonies that grew from the one center and neglecting partially overlapping colonies (examples of such colonies are indicated with the arrows in the enlarged area of Figure 3A), we quantified the colonies of each type observed in the experiments with cross-linked and de–cross-linked fragments. It turned out that observed frequencies of multicomponent colonies were nearly the same in the experiment with cross-linked chromatin fragments and control experiment with de–cross-linked DNA (compare Figure 3B and C; the raw data are presented in Supplementary Table S2). The differences in percentage of binary and ternary colonies between cross-linked and de–cross-linked samples were statistically insignificant (one-tailed unpaired *t*-test, *P* > 0.4). The low frequencies of colony colocalization were also reproduced in the experiments with longer sonication time (15-s pulse) that ensured solubilization of virtually all DNA fragments (Figure 3D and Supplementary Figure S2A, B and E and Supplementary Table S2).

**Figure 4.**
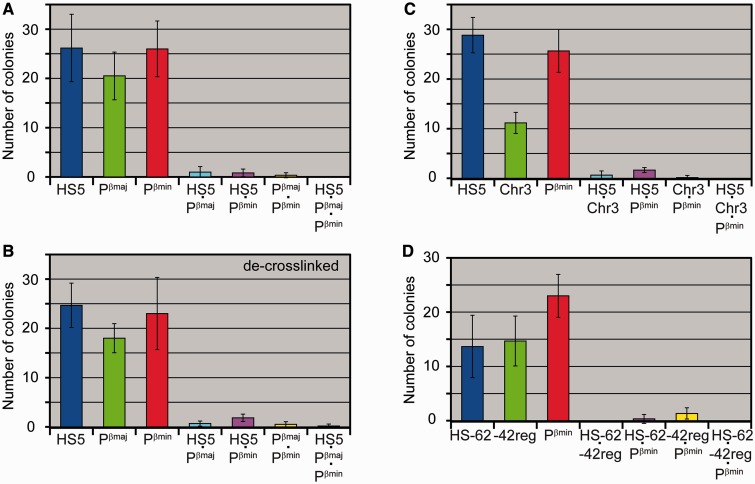
INGRID analysis of chromatin fragments released by sonication from embryonic liver cells fixed with EGS and formaldehyde. The diagrams show a mean number of colonies per gel calculated on inspection of six gels containing the material from cross-linked cells (**A, C, D**), and the same material after the cross-link reversal and DNA isolation procedure (**B**). In each case, ∼25 genomes were loaded per gel. The raw data are presented in Supplementary Table S3. All designations are as in Figure 3.

Taking into consideration the level of occasional colocalization of colonies in our experiments (∼5% for a pair of signals) and scatter of the results observed in parallel experiments, we calculated that detection of complexes containing two or more genomic fragments would be reliable (the difference between the experiment and the control would be significant) if the amount of a DNA fragment present in a complex constituted ≥3% of the total amount of this fragment. Thus, the cross-linked complexes of genomic elements under study, if existed, constituted <3% of total amount of any of these elements. One can argue that this result might be due to the low efficacy of formaldehyde cross-linking. Therefore, in a next experiment, double fixation with formaldehyde and EGS (a cross-linking agent with a longer spacer arm) was performed. In this experiment, even after 15-s pulse of sonication, the major portion of the DNA (∼70%) remained in the insoluble fraction, apparently because of the dual cross-linking, and thus was excluded from the further analysis. As in the first set of experiments, in-gel PCR was performed on soluble material with primers and molecular beacons targeting Pβ^maj^, Pβ^min^ and LCR HS5. Also, we included in the analysis the HS−62 enhancer that had been reported to participate in the assembly of β-globin ACH (17) and two ‘negative controls’: −42 region and a region from a gene desert on another chromosome (Chr3) (41) (see Figure 2A and Table 1). With the doubly cross-linked material, a notable variation in number of colonies produced from different test regions was observed. For example, HS5 and Pβ^min^ produced ∼25 colonies per gel, whereas HS−62, −42 region and Chr3 gave rise to 10–15 colonies per gel (Figure 4A, C and D and Supplementary Table S3). Partly, this variation may be due to non-equal solubilization of different fragments from the doubly cross-linked cells, as in the case of solubilization of chromatin fragments in the 3C procedure (see below). More important, with any combination of test fragments, we failed to detect any increase in the percentage of detected multicomponent colonies over the level of occasional colocalizations (Figure 4). No difference between the experiment and the control was detected (one-tailed unpaired *t*-test, *P* > 0.3).

In the last set of experiments, we prepared cross-linked chromatin fragments for the INGRID analysis according to the standard 3C protocol (17). The nuclei of formaldehyde–cross-linked embryonic liver cells were treated with SDS and HindIII restriction enzyme. After removal of the insoluble material by centrifugation, the supernatant was subjected to the INGRID analysis with primers and molecular beacons specific to the HindIII-fragments bearing LCR HS4/5, Pβ^maj^, Pβ^min^ and the test region from chromosome 3 (see earlier in the text). In accordance with our previous study (34), treatment of cross-linked nuclei with SDS and HindIII according to the standard 3C protocol resulted in the solubilization of small and non-equimolar portions of each of the fragments (Supplementary Figure S3 and Supplementary Table S4). In this experiment, the molecular colonies identified by different probes again appeared to colocalize only occasionally (Supplementary Figure S3).

All the experiments described earlier in the text were also performed with embryonic brain cells in which the globin genes are not active, and the results were similar to those obtained with erythroid cells (data not shown).

**Figure 5.**
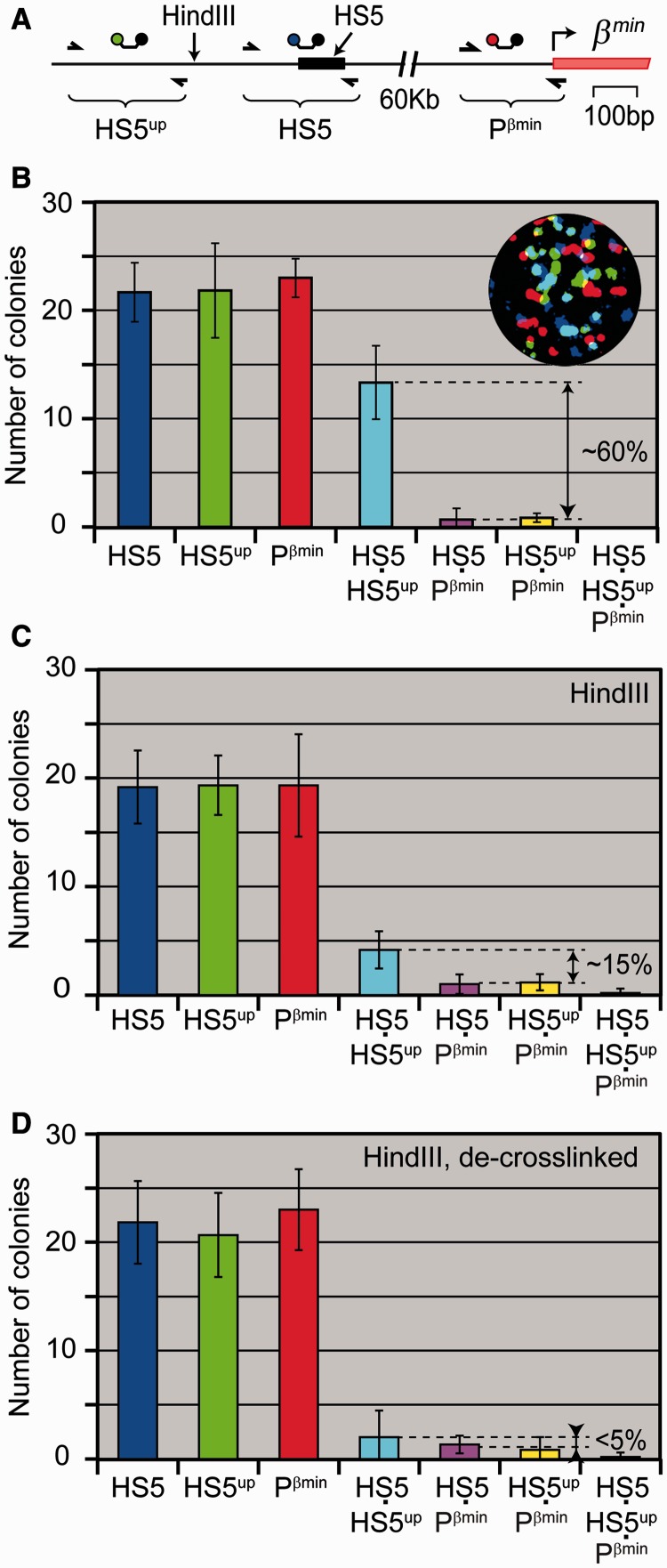
Detection of the interaction between elements that neighbor each other in the chromosomal DNA. (**A**) Map of the analyzed region showing the positions of primers (arrows) and molecular beacon probes, the HindIII restriction site, element HS5 (black bar) and the first exon of the minor β-globin gene (red bar). (**B–D**) Diagrams showing a mean number of colonies per gel and error bars (*n* = 6) in experiments with cross-linked and sonicated (7-s pulse) embryonic liver cells either not treated (B) or digested with restriction endonuclease HindIII (C) and further subjected to the cross-link reversal and DNA isolation procedure (D). In each case, an equivalent of ∼20 genomes was loaded per gel. Shown at the top-right corner of the diagram in (B) is one of the six gels run in the experiment. The percentage of binary colonies was calculated relative to the mean total number of individual colonies of each type. The raw data are presented in Supplementary Table S5. For other details see legend to Figure 3.

To ascertain if the INGRID procedure is capable of identifying chromatin elements that interact through protein bridges, we checked whether two amplicons that lie in restriction fragments that neighbor each other in the chromosomal DNA and hence should be held together by cross-linked histones would often form binary colonies on in-gel amplification of the cross-linked and digested chromatin. Into this control experiment, along with amplicons HS5 and Pβ^min^, we included a new amplicon HS5^up^ located ∼130 bp upstream of the HS5 amplicon (see Figure 5A and Table 1). After carrying out in-gel PCR on a sample that was cross-linked and sonicated, but not digested, we were able to identify ∼20 individual colonies of each type and ∼60% of the HS5 and HS5^up^ amplicons colocalized, i.e. formed binary colonies (Figure 5B). The colocalization rate was smaller than the 100% expected for amplicons that belong to the same DNA fragment produced by sonication, but this could be explained by partial damage due to sonication (see Supplementary Figure S2F) of the 650 bp sequence encompassing the two amplicons and by incomplete recovery of amplifiable DNA fragments (see Figure 2H). More important, the colocalization rate of two other amplicon pairs, HS5·Pβ^min^ and HS5^up^·Pβ^min^, was much lower, ∼3% (Figure 5B and Supplementary Table S5). After digestion of the cross-linked and sonicated chromatin at restriction site HindIII that separates the HS5 and HS5^up^ amplicons, their colocalization rate decreased, but still remained significant, ∼15% after subtraction of background (Figure 5C). When HindIII-digested material was subjected to the standard cross-link reversal and DNA isolation procedure before the in-gel PCR, the colocalization rate fell below 5% (Figure 5D), a figure close to the estimated fraction of undigested chromatin. Based on these data, we concluded that ∼10% of the neighboring restriction fragments became cross-linked during formaldehyde fixation of cells.

## DISCUSSION

Although the importance of the genome 3D structure for the regulation of gene expression is well established, the underlying mechanisms are not yet well understood (12). Based on the results of the 3C analysis, De Laat and Grosveld hypothesized that remote regulatory elements control transcription by formation of complexes with the promoters of target genes (ACH) (16). Although the ACH model has been widely accepted, it is only supported by indirect evidence as explained in ‘Introduction’ section.

ACH is thought to be assembled by means of direct interactions between proteins bound to remote elements of the genome (16). It is assumed that, on being cross-linked by formaldehyde, these complexes retain their integrity in the presence of SDS and can be extracted by SDS from nuclei treated with restriction enzymes. In a diluted solution, ligation of DNA fragments present in the same complex should be much more favorable than ligation of dispersed solitary DNA fragments. For this reason, an elevated yield of cross-ligated DNA fragments in the 3C assay is thought to reflect their proximity in the nucleus.

However, recent studies (32,42) have demonstrated that in a standard 3C assay, little, if any, DNA fragments prone to proximity ligation are extracted from cross-linked nuclei in a soluble form. The proximity ligation resulting in the generation of characteristic 3C signals appears to occur in a non-soluble cross-linked chromatin network within residual nuclei. Based on these observations, we proposed that remote regulatory elements cross-ligate with the promoters of target genes because these are held in spatial proximity by specific folding of a relatively large cross-linked chromatin domain, rather than by cross-linking of the DNA sequences through protein bridges (34). The folded chromatin domain can thus be considered as a nuclear compartment that contains regulatory elements juxtaposed with their target promoters, i.e. an expression hub (43,44). Within this nuclear compartment, interactions of genome elements may be either stable (long-living) as postulated by the ACH model, or transient and alternating.

To get further insight into the organization of regulatory complexes, it is important to determine the proportion of cells in which these complexes exist in a given moment. The results of a 3C experiment presented in relative figures cannot provide such information. Using an appropriate standard mixture of ligation junctions as an internal control, it is possible to calculate the absolute yield of the cross-ligation step (42,45). The estimates performed by two research groups showed that the yield of ligation between the fragments assumed to be present in an ACH is low, not exceeding 1% (42,45). However, the biological relevance of these observations is not clear because apart from other reasons, the proportion of a particular ligation product is the lower the higher the total number of nearby fragments available for cross-ligation, which is unknown (45,46). Therefore, it is important to have a tool for direct identification and quantification of solubilized multicomponent complexes of distant genomic elements, such as chromatin hubs, which would not rely on proximity ligation. With this aim, the INGRID protocol was developed.

In INGRID, the complexes are identified as directly as by FISH. However, as the analysis resolution can be made orders of magnitude higher (it is determined by the size of fragments into which the genome is degraded), the spatial arrangements of much less distant genomic regions can be studied. Similarly to FISH, INGRID enables estimating the proportion of genomic loci in which particular DNA sequences interact. The occurrence of a particular combination of fragments in multicomponent DNA colonies would reveal the presence of those fragments in a non-dissociable (cross-linked) complex, whereas determination of the ratio of these multicomponent colonies to all colonies that are visualized with a given probe would reveal the proportion of chromosomes in which the complex is present.

In the present study, we used the INGRID protocol to analyze the putative ACH of the mouse β-globin locus in erythroid cells. In this classical model system, we failed to detect complexes bearing the promoters of the major and minor β-globin genes and HS5 of the LCR (either in triads or in pairs) (Figure 3). Taking into account the background due to occasional colocalization of molecular colonies, we concluded that, if existed, such complexes would occur in <3% of chromosomes present in the analyzed sample. The capability of the INGRID approach of detecting protein-mediated interactions in the chromatin was demonstrated by the finding that amplicons from separate restriction fragments did form binary molecular colonies at a rate of ∼10% if those fragments neighbored each other in the chromosome (Figure 5C). The latter value is ∼6 times higher than the frequency of ligation of neighboring DNA fragments in the 3C procedure (∼1.5%) that was estimated in our previous study (45). The discrepancy can be easily explained by the availability of multiple targets for proximity ligation.

Taken together, the results of the present study suggest that ACHs of the type proposed by De Laat and Grosveld (16) either do not exist or are unstable and/or short-living so that in a given moment they are assembled only in a minor fraction of cells present in a population. This conclusion may seem contradictory to the FISH results that in some instances genes and remote regulatory elements are colocalized in a significant proportion of cells in a population, which may be as high as 30% (47) or even 85% (48). However, FISH cannot discriminate between the elements that interact directly [ACH model (16)] and those that just occur in one expression compartment [expression hub model (12,44)]. Our results do not question the importance of the promoter–enhancer interactions but rather suggest a more flexible mode of these interactions than is postulated by the ACH model.

Of course, the possibility that complexes existing in living cells are poorly fixed by formaldehyde should also be considered. To address such a possibility, we used EGS, a cross-linking agent with a longer spacer arm in addition to the formaldehyde, as has been proposed for fixation of multiprotein complexes that are not directly bound to DNA (49,50). Yet, in this case too, we failed to detect complexes between the promoters and enhancers of the β-globin genes in the material solubilized from erythroid cells.

Obviously, suitability of the INGRID protocol for analyzing the chromosome folding entirely relies on solubility of the analyzed sample. Non-solubilized nuclei or large clamps of chromatin would colocalize a number of fragments irrespective of their presence in specific complexes, and hence must be removed from the solution applied to the gel. As mentioned previously, the major part of cross-linked material is not solubilized from nuclei even after SDS extraction and treatment with a restriction endonuclease according to a standard 3C protocol (34,42). Therefore, before spreading over the gels, the samples were sonicated under conditions that ensure the integrity of test amplicons and solubilization of >90% DNA (Supplementary Figure S2).

In conclusion, we would like to note that the INGRID assay might become an important tool for studying the 3D structure of the genome. The small amounts of the starting material required by the reported protocol enable studies of individual cells. Although here, only three amplicons were monitored to visualize ternary interactions, the INGRID protocol can readily be upgraded to analyzing four or more amplicons by using a device capable of simultaneous monitoring a greater number of different fluorophores. Using longer amplicons and/or denser gels could reduce the size of molecular colonies down to <10 µm, thereby allowing thousands of them to be resolved on a single gel and greatly increasing throughput of the assay (33). By determining the composition and quantity of associated genomic elements, the INGRID assay could be useful in studies on both the diversity and dynamics of any chromatin structures that are available in a soluble form.

## SUPPLEMENTARY DATA

Supplementary Data are available at NAR Online.

## FUNDING

Presidium of the Russian Academy of Sciences (grants from Molecular and Cellular Biology Program to A.A.G., A.B.C. and S.V.R.); Russian Foundation for Basic Research [11-04-00361-а, 11-04-91334-NNIO_а, 12-04-00036-а, 12-04-33040, 12-04-00698, 12-04-93109_CNRS and 13-04-00245]; President of the Russian Federation for young scientists [MK-3813.2012.4] and Dmitri Zimin’s foundation ‘Dynasty’. Funding for open access charge: Molecular and Cellular Biology Program of the Presidium of the Russian Academy of Sciences.

*Conflict of interest statement.* A.B.C. and H.V.C. are co-inventors and co-owners of a patent (RU2394915) and patent applications (WO2007111639, US2009105082, EP1999268) disclosing the real time detection of growing molecular colonies. A.A.G., E.S.C. and S.V.R. declare no competing financial interests.

## Supplementary Material

Supplementary Data
