# Supplementary material for: Quantitative analysis of genomic element interactions by molecular colony technique
Source: Nucleic Acids Res. 2013 Dec 24;42(5):e36. doi: 10.1093/nar/gkt1322 (PMC3950710; doi:10.1093/nar/gkt1322)
Supplement: Supplementary Data [file supp_42_5_e36__index.html]

Quantitative analysis of genomic element interactions by molecular colony technique — Supplementary Data 

# Quantitative analysis of genomic element interactions by molecular colony technique

## Supplementary Data

files

**Files in this Data Supplement:**

- Supplementary Data - pdf file
