# Supplementary material for: Quantitative analysis of genomic element interactions by molecular colony technique
Source: Nucleic Acids Res. 2013 Dec 24;42(5):e36. doi: 10.1093/nar/gkt1322 (PMC3950710; doi:10.1093/nar/gkt1322)
Supplement: Supplementary Data [file supp_gkt1322_nar-02532-met-h-2013-File007.pdf]

SUPPLEMENTARY TABLES

Table S1. Sequences of primers and TaqMan probes used to estimate the quantities of DNA fragments. S, sense primers; A/S, antisense primers (in regard to the direction of transcription of the β-globin genes); TM, TaqMan probes.

Test-region	Amplicon size, bp	Primer/TaqMan set (5'-3')
-42 region short	163	S TATCCCGTGACTATACCTTAATGAA<br>A/S GCCAATGTGATGAGTGCAGAG<br>TM FAM-CTTGTGGAGAA(T-BHQ1)GTCATAGACTATACGAGTGAA-PO4
-42 region long	578	S TATCCCGTGACTATACCTTAATGAA<br>A/S TGTTTAAGCATTTAGAAAGAAACTGAA<br>TM FAM-CTTGTGGAGAA(T-BHQ1)GTCATAGACTATACGAGTGAA-PO4
HS5	117	S TACAAGTCTCCTCATGTTCCCAA<br>A/S TTTTCAGACATACTCCTCTATCCAG<br>TM FAM-TAGCCTCAGT(T-BHQ1)ACCCGACATTAGTTCTTAGT-PO4
upstream of $P\beta^{maj}$	532	S AAAATTGAATAGAATCACTACTGCAC<br>A/S ACTGCCTTCAGAGAATCACCCT<br>TM FAM-AATAAGGTACA(T-BHQ1)TTGAATTGAGCATACCCTG-PO4
$P\beta^{maj}$ short	163	S TCAGTAGTTGATTGAGCAAATGTGT<br>A/S TCGGTGATGACAAGCATATTTCT<br>TM FAM-TCCCTCTGAA(T-BHQ1)AATGTTTGTCCTTATCTGTG-PO4
$P\beta^{maj}$ long	293	S TCAGTAGTTGATTGAGCAAATGTGTT<br>A/S CTATGTCAGAAGCAAATGTGAGGAG<br>TM FAM-TCCCTCTGAA(T-BHQ1)AATGTTTGTCCTTATCTGTG-PO4
β^{maj}/β^{min} short	114	S AATCGCTGCTCCCCCTCACT<br>A/S GATGGGAAGTAAATAACCAGCTTAAT<br>TM FAM-ACCAAAGAAAGAGGAAA(T-BHQ1)GACAACACAGAACA-PO4
β^{maj}/β^{min} long	263	S AGTCTGATGGGCACCTCCTG<br>A/S GATGGGAAGTAAATAACCAGCTTAAT<br>TM FAM-ACCAAAGAAAGAGGAAA(T-BHQ1)GACAACACAGAACA-PO4
OR69	110	S GGCAAAGCACATTTACCTGAT<br>A/S ACACTTGTTCCCTGAGATGGTT<br>TM FAM-TCCACCCTCA(T-BHQ1)AGCATCCTCATAGCAT-PO4

Table S2. Raw data of the INGRID analysis of chromatin fragments released by sonication from formaldehyde-fixed Ter119+ cells (linked to Figure 3).

Ter119+ 7-second sonication

gel#	HS5	Pmaj	Pmin	HS5▪Pmaj	HS5▪Pmin	Pmaj▪Pmin	HS5▪Pmaj▪Pmin
1	26	25	23	2	0	0	0
2	21	19	28	1	2	0	0
3	29	19	25	1	3	1	0
4	35	17	33	2	0	0	0
5	29	22	24	0	0	1	1
6	29	28	23	2	0	0	0
**Mean**	**28,2**	**21,7**	**26,0**	**1,3**	**0,8**	**0,3**	**0,2**
**SD**	**4,6**	**4,2**	**3,9**	**0,8**	**1,3**	**0,5**	**0,4**

Ter119+ 7-second sonication, de-crosslinked

gel#	HS5	Pmaj	Pmin	HS5▪Pmaj	HS5▪Pmin	Pmaj▪Pmin	HS5▪Pmaj▪Pmin
1	32	17	25	1	0	0	0
2	28	24	36	1	1	3	0
3	40	36	35	2	2	1	1
4	35	24	38	2	2	1	0
5	26	29	34	2	0	1	0
6	22	25	36	1	2	1	0
**Mean**	**30,5**	**25,8**	**34,0**	**1,5**	**1,2**	**1,2**	**0,2**
**SD**	**6,5**	**6,3**	**4,6**	**0,5**	**1,0**	**1,0**	**0,4**

Ter119+ 15-second sonication

gel#	HS5	Pmaj	Pmin	HS5▪Pmaj	HS5▪Pmin	Pmaj▪Pmin	HS5▪Pmaj▪Pmin
1	38	24	36	0	3	0	0
2	26	28	25	0	4	1	0
3	34	25	33	1	2	1	0
4	34	33	44	3	3	2	0
5	37	29	35	2	1	0	0
6	27	31	27	0	0	1	0
7	44	48	31	5	2	4	0
8	27	25	30	1	3	0	0
9	28	25	24	2	3	2	0
**Mean**	**32,8**	**29,8**	**31,7**	**1,6**	**2,3**	**1,2**	**0,0**
**SD**	**6,2**	**7,5**	**6,2**	**1,7**	**1,2**	**1,3**	**0,0**

Table S3. Raw data of the INGRID analysis of chromatin fragments released by sonication from embryonic liver cells fixed with EGS and formaldehyde (linked to Figure 4).

Liver

gel#	HS5	Pmaj	Pmin	HS5▪Pmaj	HS5▪Pmin	Pmaj▪Pmin	HS5▪Pmaj▪Pmin
1	16	26	28	1	0	0	0

2	33	13	18	1	1	0	0
3	23	22	25	3	0	1	0
4	32	19	35	0	1	0	0
5	31	25	23	1	2	0	0
6	22	18	27	0	1	1	0
**Mean**	**26,2**	**20,5**	**26,0**	**1,0**	**0,8**	**0,3**	**0,0**
**SD**	**6,9**	**4,8**	**5,7**	**1,1**	**0,8**	**0,5**	**0,0**

Liver, de-crosslinked

gel#	HS5	Pmaj	Pmin	HS5▪Pmaj	HS5▪Pmin	Pmaj▪Pmin	HS5▪Pmaj▪Pmin
1	21	15	20	1	2	1	0
2	28	15	30	1	2	0	1
3	23	16	16	0	1	0	0
4	19	20	21	0	1	1	0
5	31	21	34	1	3	0	0
6	26	21	17	1	2	1	0
**Mean**	**24,7**	**18,0**	**23,0**	**0,7**	**1,8**	**0,5**	**0,2**
**SD**	**4,5**	**3,0**	**7,3**	**0,5**	**0,8**	**0,5**	**0,4**

Liver

gel#	HS5	Chr3	Pmin	HS5▪Chr3	HS5▪Pmin	Chr3▪Pmin	HS5▪Chr3▪Pmin
1	27	12	30	1	2	0	0
2	34	9	21	0	2	0	0
3	32	13	30	1	1	1	0
4	25	13	26	0	2	0	0
5	26	8	27	0	2	0	0
6	29	12	20	2	1	0	0
**Mean**	**28,8**	**11,2**	**25,7**	**0,7**	**1,7**	**0,2**	**0,0**
**SD**	**3,5**	**2,1**	**4,3**	**0,8**	**0,5**	**0,4**	**0,0**

Liver

gel#	HS-62	-42reg	Pmin	HS-62▪-42reg	HS-62▪Pmin	-42reg▪Pmin	HS-62▪-42reg▪Pmin
1	11	19	29	0	0	2	0
2	22	16	27	0	2	2	0
3	10	9	21	0	0	0	0
4	10	19	20	0	0	2	0
5	9	16	21	0	0	2	0
6	20	9	20	0	0	0	0
**Mean**	**13,7**	**14,7**	**23,0**	**0,0**	**0,3**	**1,3**	**0,0**
**SD**	**5,8**	**4,6**	**3,9**	**0,0**	**0,8**	**1,0**	**0,0**

Table S4. Raw data of the INGRID analysis of chromatin fragments released from formaldehyde-fixed embryonic liver cells using the 3C protocol (linked to Figure S3).

Liver

gel#	HS4/5	Pmaj	Pmin	HS4/5▪Pmaj	HS4/5▪Pmin	Pmaj▪Pmin	HS4/5▪Pmaj▪Pmin
1	9	61	18	1	0	4	0
2	12	67	24	1	0	2	1
3	6	70	20	0	0	3	0
4	6	70	21	1	0	2	1
5	13	92	23	2	0	6	0
6	14	66	22	1	0	3	0
**Mean**	**10,0**	**71,0**	**21,3**	**1,0**	**0,0**	**3,3**	**0,3**
**SD**	**3,5**	**10,8**	**2,2**	**0,6**	**0,0**	**1,5**	**0,5**

Liver

gel#	HS4/5	Chr3	Pmin	HS4/5▪Chr3	HS4/5▪Pmin	Chr3▪Pmin	HS4/5▪Chr3▪Pmin
1	12	7	23	0	0	1	0
2	14	12	28	0	0	0	0
3	10	9	23	0	1	1	0
4	11	6	13	1	1	0	0
5	21	17	25	1	1	1	0
6	7	8	28	0	1	0	0
**Mean**	**12,5**	**9,8**	**23,3**	**0,3**	**0,7**	**0,5**	**0,0**
**SD**	**4,8**	**4,1**	**5,5**	**0,5**	**0,5**	**0,5**	**0,0**

Table S5. Raw data of the INGRID analysis of test-amplicons located close to each other in DNA sequence (linked to Figure 5).

Liver, untreated

gel#	HS5	HS5up	Pmin	HS5▪HS5up	HS5▪Pmin	HS5up▪Pmin	HS5▪HS5up▪Pmin
1	26	28	22	16	0	1	0
2	19	21	24	11	0	1	0
3	21	18	23	11	2	1	0
4	24	26	20	19	0	0	0
5	20	21	24	12	0	1	0
6	20	17	25	11	2	1	0
**Mean**	**21,7**	**21,8**	**23,0**	**13,3**	**0,7**	**0,8**	**0,0**
**SD**	**2,7**	**4,4**	**1,8**	**3,4**	**1,0**	**0,4**	**0,0**

Liver, HindIII digested

gel#	HS5	HS5up	Pmin	HS5▪HS5up	HS5▪Pmin	HS5up▪Pmin	HS5▪HS5up▪Pmin
1	16	23	21	4	1	1	0
2	22	20	22	4	2	1	0
3	20	18	12	5	0	2	0
4	24	21	22	7	1	2	0
5	17	15	24	3	2	0	1
6	16	19	15	2	0	1	0
**Mean**	**19,2**	**19,3**	**19,3**	**4,2**	**1,0**	**1,2**	**0,2**
**SD**	**3,4**	**2,7**	**4,7**	**1,7**	**0,9**	**0,8**	**0,4**

Liver, HindIII, de-crosslinked

gel#	HS5	HS5up	Pmin	HS5▪HS5up	HS5▪Pmin	HS5up▪Pmin	HS5▪HS5up▪Pmin
1	19	24	24	0	2	0	0
2	25	26	28	0	2	3	0
3	20	15	22	3	1	1	0
4	27	20	26	6	2	0	0
5	23	20	20	3	1	1	1
6	17	19	18	0	0	0	0
**Mean**	**21,8**	**20,7**	**23,0**	**2,0**	**1,3**	**0,8**	**0,2**
**SD**	**3,8**	**3,9**	**3,7**	**2,4**	**0,8**	**1,2**	**0,4**

SUPPLEMENTARY FIGURE LEGENDS

Figure S1. Agarose gel electrophoresis of PCR products obtained with indicated primer pairs (**A**) or their combinations (**B**) in a liquid PCR using mouse genomic DNA as a matrix for amplification and 25, 30, 35 or 40 cycles of amplification (from left to right). The sizes of PCR products (bp) are shown in parentheses. Asterisks indicate primer combinations used in the molecular colony analysis. The sequences of primers are presented in Table S1. DNA size marker – Fermentas, SM0331.

Figure S2. Solubilization and breakage of β-globin gene domain fragments bearing the selected test-amplicons upon sonication of formaldehyde-fixed mouse embryonic liver cells. (**A**) Partitioning of total DNA between soluble (supernatant) and insoluble (pellet) fraction upon sonication for indicated time and size distribution of DNA fragments. After sonication, the soluble and insoluble fractions were separated by centrifugation followed by DNA isolation and electrophoretic separation (agarose gel, ethidium bromide staining, DNA size marker – Fermentas, SM0331). (**B**) Relative amounts of DNA in soluble and insoluble fractions, as determined by fluorometric assays (Qubit, Invitrogen). In each experiment, the total amount of DNA in two fractions is set as 100. (**C**) Map of the murine β-globin locus (all designations are as in Figure 2A) showing the position of INGRID test-amplicons (black boxes below the map) and qPCR test-amplicons (colored boxes). (**D,E**) Graphs showing the degree of solubilization of different test-amplicons (S) – a ratio of the total amount of a test-amplicon in the soluble fraction (including broken and unbroken amplicons) to the total amount of this amplicon in two fractions, which was calculated based on the results of qPCR analysis of purified DNA templates using the following equation: S(%)=[A(total)–A(debris)]/A(total)*100, where A(total) – PCR signal observed with a crosslinked non-sonicated non-fractionated control template, A(debris) – PCR

signal observed in the insoluble fraction. (**F,G**) Graphs showing the level of undamaged test-amplicons in the soluble fraction (I) – a ratio of the amount of an unbroken test-amplicon in the soluble fraction to the total amount of this amplicon in the soluble fraction, which was calculated using the following equation: I(%)=A(super)/[A(total)–A(debris)]*100, where A(super) – PCR signal observed in the soluble fraction. Based on the results of electrophoresis (**A**), it is assumed in our calculations that test-amplicons in the insoluble fraction are for the most past unbroken. Graphs in (**D,F**) show the results of experiments with 7-second sonication, graphs in (**E,G**) – 15-second sonication. To avoid the differences in the efficacy of amplification on short and long DNA templates, before PCR all samples were treated with a restriction enzyme cutting outside of a chosen test-amplicon. The sequences of primers and TaqMan probes used for qPCR are presented in Table S1. On each graph, the results for individual amplicons are presented in respect to amplicon size and using the same color code as assigned in (**C**) to show the amplicon positions. Gray shading at the background of the graphs shows the size interval in which all INGRID test-amplicons fall. The blue lines are trend lines. Error bars represent the standard deviation for two independent experiments.

Figure S3. INGRID analysis of chromatin fragments released from formaldehyde-fixed embryonic liver cells using the 3C protocol. The diagrams show a mean number of colonies per gel calculated upon inspection of 6 gels containing the material from crosslinked cells. The raw data are presented in Table S4. All designations are as in Figure 3.

SUPPLEMENTARY FIGURES

Figure S1

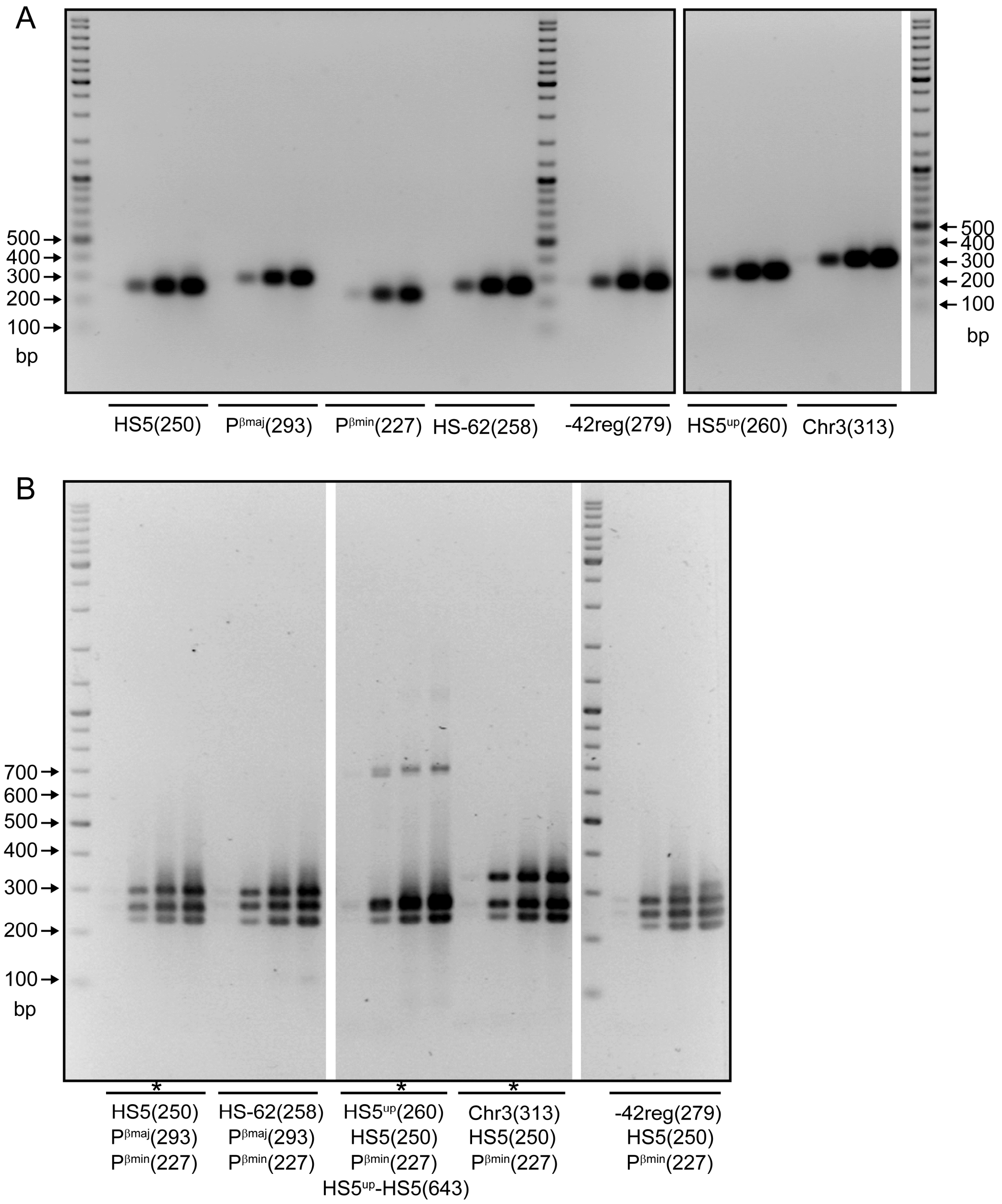

Figure S2

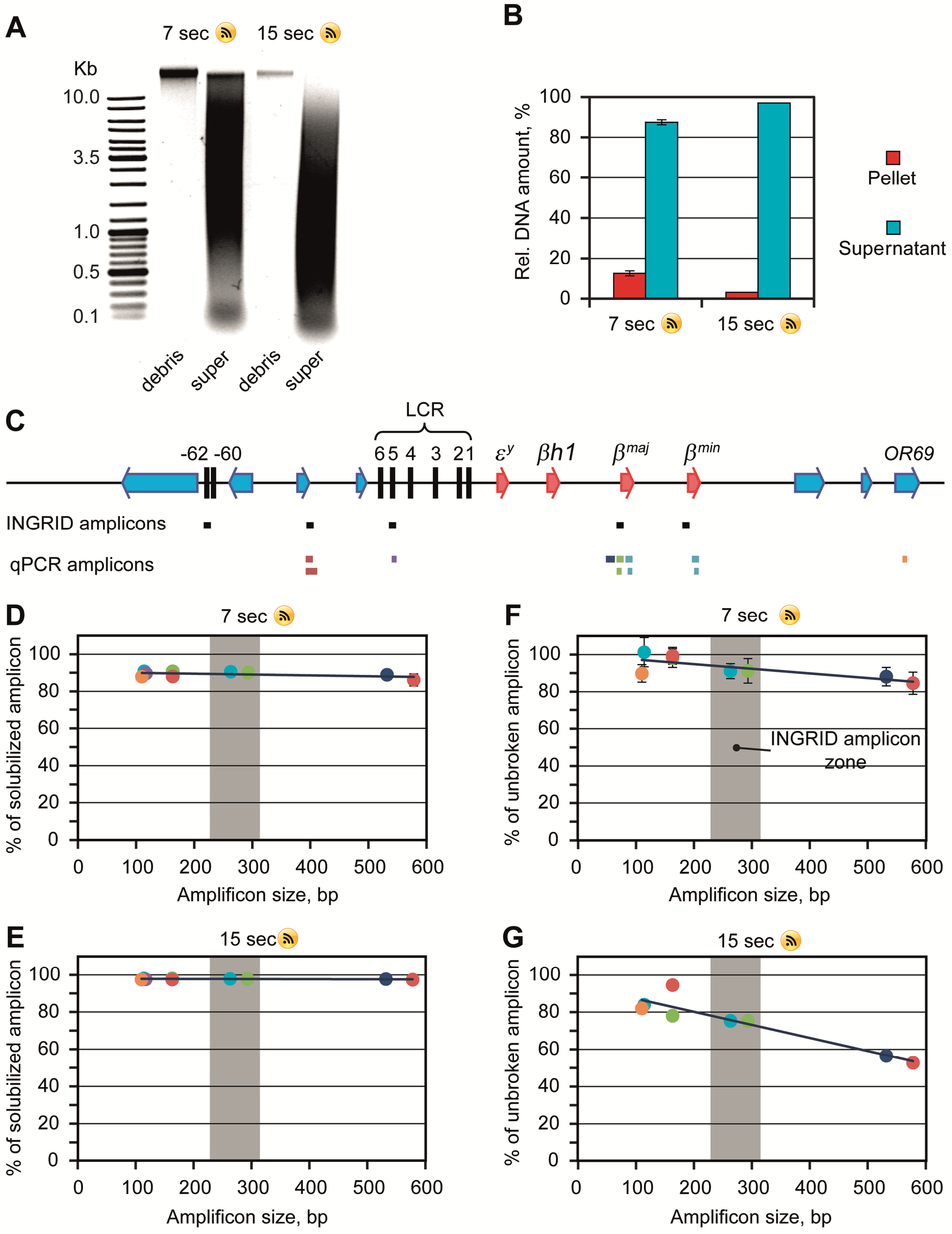
A
7 sec
15 sec
Kb
10.0
3.5
1.0
0.5
0.1
debris
super
debris
super
B
Rel. DNA amount, %
100
80
60
40
20
0
7 sec
15 sec
Pellet
Supernatant
C
LCR
-62 -60
6 5 4 3 2 1
εy
βh1
βmaj
βmin
OR69
INGRID amplicons
qPCR amplicons
D
7 sec
% of solubilized amplicon
Amplicon size, bp
E
15 sec
% of solubilized amplicon
Amplicon size, bp
F
7 sec
% of unbroken amplicon
INGRID amplicon zone
Amplicon size, bp
G
15 sec
% of unbroken amplicon
Amplicon size, bp

Figure S3

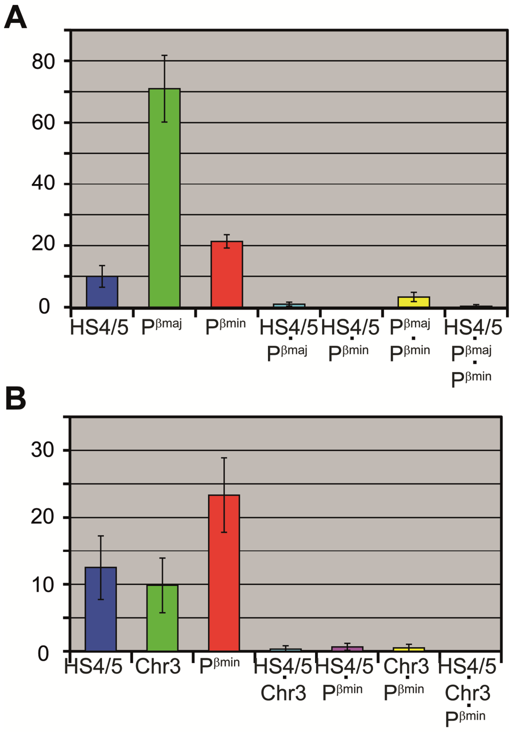
A
80
60
40
20
0
HS4/5
Pβmaj
Pβmin
HS4/5 : Pβmaj
HS4/5 : Pβmin
Pβmaj : Pβmin
HS4/5 : Pβmaj : Pβmin
B
30
20
10
0
HS4/5
Chr3
Pβmin
HS4/5 : Chr3
HS4/5 : Pβmin
Chr3 : Pβmin
HS4/5 : Chr3 : Pβmin
